# Key Cell Types and Biomarkers in Heart Failure Identified through Analysis of Single-Cell and Bulk RNA Sequencing Data

**DOI:** 10.1155/2023/8384882

**Published:** 2023-12-26

**Authors:** Ying Kong, Ning Yang, Zhiqing Luo, Ruiting Huang, Quhuan Li

**Affiliations:** ^1^School of Biology and Biological Engineering, South China University of Technology, Guangzhou 510006, Guangdong, China; ^2^Guangdong Provincial Engineering and Technology Research Center of Biopharmaceuticals, South China University of Technology, Guangzhou 510006, Guangdong, China

## Abstract

Heart failure (HF) is a complex clinical syndrome resulting from various cardiac diseases and a significant medical issue worldwide. Although the role of inflammation in HF pathogenesis is well-known, the specific cell types and regulatory molecules involved remain poorly understood. Here, we identified key cell types and novel biomarkers via an analysis of single-cell and bulk RNA sequencing data obtained from patients with two major HF types of ischemic cardiomyopathy and dilated cardiomyopathy. Myeloid cells were identified as the primary cell population involved in HF through cellular fraction and gene set enrichment analysis. Additionally, differential analysis of myeloid cells revealed crosstalk between cellular communication and cytokine-regulated immune responses in HF, with the MIF pathway emerging as a crucial immune regulatory pathway. The CD74/CXCR4 receptor complex in myeloid cell subgroup M*φ*2 was significantly upregulated, potentially acting as a crucial regulator in HF. Upon receiving the MIF signal molecule, the CD74/CXCR4 receptor can activate NF-*κ*B signaling to produce chemokines and thereby enhance the inflammatory response. CD74 and CXCR4 may serve as biomarkers and treatment targets for HF.

## 1. Introduction

Heart failure (HF) is a complex clinical syndrome that impairs cardiac function, leading to suboptimal blood pumping capacity to satisfy the body's metabolic needs [1]. It represents a significant public health concern, with over 64 million individuals affected worldwide [2, 3]. The aging population also contributes to the annual increase in HF prevalence, with a projected 46% increase in incidence by 2030 [4]. HF is typically the terminal stage of multiple cardiovascular diseases and may be triggered by various cardiac diseases, with ischemic cardiomyopathy (ICM) and dilated cardiomyopathy (DCM) being the leading causes [3, 5]. Patients with HF receive individualized and precise treatment strategies based on the classification of the left ventricular ejection fraction (LVEF) [6]. This requires a deeper understanding of the pathophysiology of HF and associated transcriptomic and genetic mechanisms.

Earlier research has suggested that imbalanced inflammation plays a crucial role in the pathophysiology of HF [7, 8], with monocytes and macrophages playing essential roles in maintaining heart homeostasis and immune defense [9]. Macrophages may polarize into the M1 phenotype with proinflammatory capabilities or the M2 phenotype with anti-inflammatory functions [10, 11]. Poor left ventricular (LV) remodeling in patients due to pressure overload requires cardiac macrophages stemming from CCR2^+^ monocytes [12]. These reports indicate that functionally heterogeneous macrophage subsets exist in the immune microenvironment of the heart and contribute to HF development. However, the specific mechanisms and macrophage subpopulations involved are not yet clear.

Single-cell RNA sequencing (scRNA-seq) makes it possible to examine the cell populations participating in HF at the molecular level and to investigate how the interaction between different cell subsets is regulated by ligand–receptor (L–R) [13]. Studies on the cardiovascular immune microenvironment in mice with pressure overload have shown that immune activation involves several cell types [14]. For instance, CD72^hi^ macrophages release proinflammatory factors that lead to heart damage, suggesting that targeting CD72^hi^ macrophages could be a novel therapeutic strategy for HF [15]. A high-resolution single-cell landscape was also constructed for patients with HF to analyze immune cell populations [16], yet with a small sample size, which limited the scope of the analysis. Here, through a joint analysis of both scRNA-seq and bulk RNA-seq data, we elucidated key cell types and cell type-specific genes that play important roles in the pathogenesis of HF.

**Figure 1:**
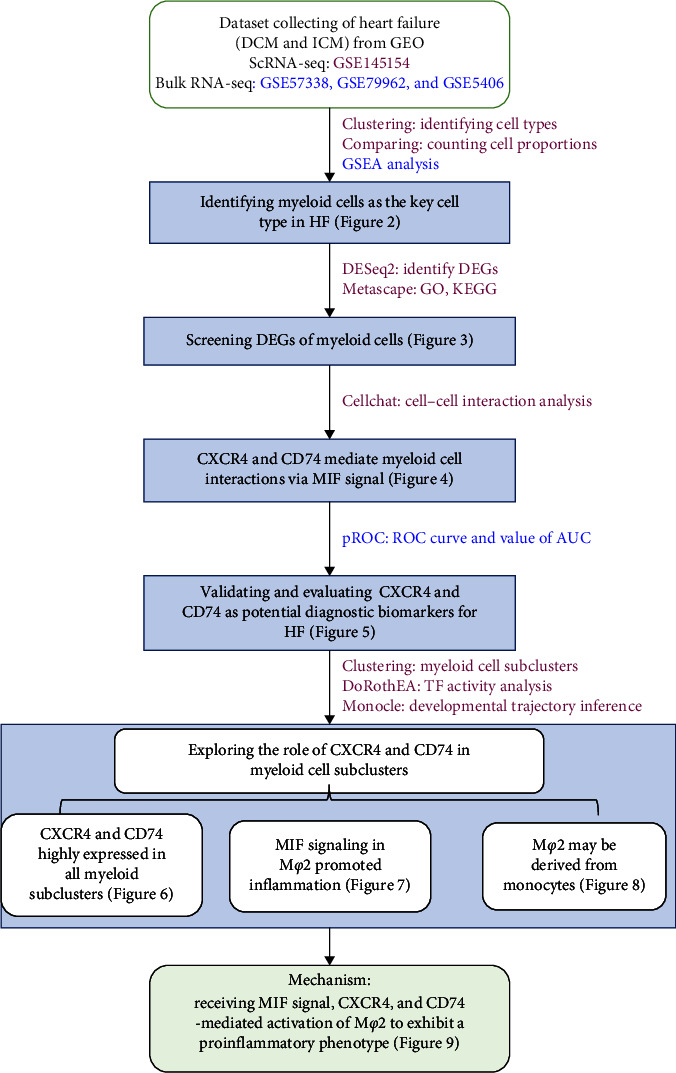
The overall research framework.

We conducted a bioinformatics analysis of HF samples comprising ICM and DCM (Figure 1) to explore key cell types involved in HF and identify potential biomarkers. First, we identified myeloid cells involved in HF through cluster analysis in scRNA-seq data to identify cell types, comparing cellular fractions between different groups, and conducting gene set enrichment analysis (GSEA) using bulk RNA-seq data. We then analyzed the differential expression of myeloid cells across the groups and explored the function of these genes with differential expression using Metascape. Further analysis of cellular interaction revealed that molecules within the MIF signaling pathway were significantly upregulated in HF and could potentially serve as valuable biomarkers. Finally, we reclustered myeloid cells at a higher resolution to study the heterogeneity of the myeloid cell subtypes and their potential differentiation relationships.

## 2. Materials and Methods

### 2.1. Data Collection

Four datasets included in this study were identified by searching using the following keywords: HF, DCM, and ICM. One single-cell RNA-seq dataset (GSE145154) from heart and blood contained one normal, two DCM, and three ICM samples [16]. The three bulk RNA-Seq datasets included normal, DCM, and ICM samples: GSE79962 [17], GSE5406 [18], and GSE57338 [19]. GSE141910 [20], which includes DCM and normal samples with recorded LVEF information, was used for further analysis and validation. A summary of the datasets is presented in Table 1, with all data sourced from the Gene Expression Omnibus (GEO) database (https://www.ncbi.nlm.nih.gov/geo). For bulk RNA-Seq data, the normal groups in GSE5406, GSE57338, and GSE79962 consisted of normal LV myocardial tissue ejection fractions of 56% ± 7%, LV-free wall tissue obtained from unused donor hearts, and hearts from nonfailure donors that were considered unfit for heart transplantation. The normal group in the single-cell dataset, GSE145154, was from the left ventricle, with an ejection fraction of 65%. In summary, the normal group in the bulk dataset was derived from the LV tissue of heart donors without HF. For the single-cell dataset, the normal group was derived from healthy individuals. The normal group was defined as individuals with EF ≥ 50% and no history of HF.

### 2.2. ScRNA-Seq Analysis

After standard data preprocessing and quality control measures, the scRNA-seq data underwent cell clustering analysis using the R package Seurat [21]. Cells expressing less than 500 genes, more than 10% of mitochondrial genes, or cells with UMI count of less than 800 or more than 8,000 were excluded as low-quality cells. After filtering, 29,382 cells were retained for the subsequent analysis. LogNormalize was used to standardize the expression data, FindVariableFeatures with the dispersion method was employed to identify the top 2,000 features with high variability, FindIntegration was utilized to detect anchors with default conditions, and IntegrateData was applied to integrate objects with the top 30 dimensions. The integrated dataset was then normalized using Seurat's normalized data and scale data functions. Finally, cells were clustered at 0.5 resolution using the FindClusters function and subsequently visualized using 2D uniform manifold approximation and projection (UMAP).

The FindAllMarkers function was utilized to detect marker genes for each cluster, with a minimum percentage of expression value set to 0.3 and a log-fold change threshold set to 0.6. Subsequently, cell types within each cluster were defined by comparing the marker genes with signature genes from published research [16] and the public CellMarker database (http://bio-bigdata.hrbmu.edu.cn/CellMarker). Clusters containing markers for both cell types were removed. Myeloid cells were divided into subgroups after grouping and separation using the subset commands.

### 2.3. Differential Gene Expression Analysis

Owing to the high percentage of false positives in single-cell differential expression analysis, which treats each cell as a biological replication, a pseudobulk approach was adopted to conduct intergroup differential gene analysis [22–24]. DESeq2 [25] was used for differential expression analyses of single-cell expression profiles. For myeloid cells with scRNA-seq data, the reads for transcriptional replication were first aggregated and converted from the gene-cell matrix to a gene-replication matrix using matrix multiplication. DEGs were visualized using the ggplot2 package. For bulk RNA-seq data, DEGs for microarray expression profile data were identified using the limma package [26]. Only genes with a |log2FC| > 1 and an adjusted *p*-value of less than 0.05 were deemed to be DEGs.

### 2.4. Gene Set Enrichment Analysis

To uncover the molecular basis of HF, DEGs obtained from DCM/ICM samples were used to identify markedly changed cell types. For this purpose, GSEA [27] was performed using clusterProfiler package [28] using cell-specific marker genes, which allowed us to determine the degree of cellular enrichment. In addition, GSEA was also conducted to reveal the enrichment features of the cell clusters, using a reference gene set selected from the MSigDB database, focusing on gene sets for biological processes (BPs).

### 2.5. Function Enrichment Analysis

DEGs identified using the pseudobulk method were uploaded to Metascape (https://metascape.org/) for BP enrichment analysis. Differentially expressed genes (p < 0.01) in each myeloid subcluster were identified using the FindMarkers function of the Seurat package, employing the MAST method. After transforming gene symbols to Entrez gene IDs, gene ontology (GO) was conducted using the ClusterProfiler package and org.Hs.eg.db package. Proinflammatory and anti-inflammatory scores were obtained using the AddModuleScore function in Seurat. Specifically, 10 proinflammatory-related genes (IL1B, TNF, CCL2, CCL3, CCL5, CCL7, CCL8, CCL13, CCL17, and CCL22) were used to calculate proinflammatory scores, whereas nine anti-inflammatory-related genes (IL1RN, IL10, IL4, IL11, IL1R2, TGFB1, TNFRSF1A, TNFRSF1B, and IL18BP) were used to derive anti-inflammatory scores.

### 2.6. Cell–Cell Communication Analysis

To shed light on the cellular communication network among various cell clusters, CellChat [29] was employed to deduce and characterize possible cell–cell interactions. Briefly, the normalized scRNA-seq data were first split into DCM, ICM, and normal groups, and then CellChat objects for each group were generated separately to calculate cell communication networks. Finally, the three groups of CellChat objects were combined. To identify the upregulated ligand–receptor pairs, myeloid cells were defined as receivers or senders to compare the interactions between the normal control and DCM/ICM groups.

### 2.7. Transcription Factor Activity Analysis

DoRothEA [30] was used to assess transcription factor (TF) activity for myeloid subsets from the scRNA-seq data. Transcription factor regulatory networks that exhibited higher confidence levels (from A to C) supported by evidence were extracted for subsequent analyses. The scale method was used to measure the viper scores. Fifteen TFs with the greatest variations were identified and are displayed.

### 2.8. Developmental Trajectory Inference

Monocle3 [31] and Monocle2 [32] were employed to order the cells based on pseudotime analysis. First, the normalized gene expression matrix within Seurat as import for Monocle3. Then, a CellDataSet object was created using the new_cell_data_set function of Monocle3 and handled by the preprocess_cds function using the default settings. Data dimensionality was reduced using the reduced-dimension function with preprocess_method set to principal component analysis and reduction_method set to UMAP. The trajectory and order of the cells were learned. Ordered cells were visualized using the cell plot function.

A similar approach was used by Monocle2. The cell clusters with potential relationships were extracted using the Seurat subset command. The CellDataSet objects were created using, as described in. CellDataSet function in monocle2. The genes were filtered out if their average expression level was below 0.1 and less than 10 cells. Following the calculated scale factors and estimated dispersions, the variably expressed genes between clusters along the trajectory were defined using a differential gene test function. After cell ordering, visualization was performed using the plot_cell_trajectory and plot_genes_in_pseudotime functions.

### 2.9. Statistical Analysis

Student's *t*-test was conducted to determine the significance of gene expression differences between groups (DCM vs. normal, ICM vs. normal). Multiple testing was performed by adjusting *p* values to *q* values using the Benjamini–Hochberg method. In addition, receiver operating characteristic (ROC) curve analysis was performed using pROC [33], which allowed us to assess the sensitivity and specificity of each gene according to its expression level. The area under the ROC curve (AUC) was calculated as a measure of performance. Spearman's correlation was used to analyze the correlation between gene expression and LVEF. Statistical significance was defined as a *p*-value or *q*-value less than 0.05.

## 3. Results and Discussion

### 3.1. ScRNA-Seq Profiles Revealed the Heterogeneity of Immune Cells in HF Patients

**Figure 2:**
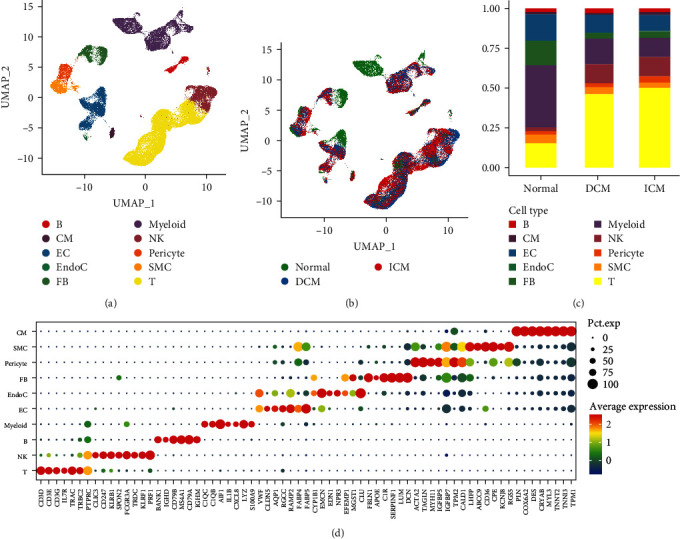
Overview of cell populations was profiled for healthy donors and those with failing hearts: (a) the UMAP plot of scRNA-seq data depicts 10 distinct cell types; (b) the UMAP plot of cells grouped by color for DCM, ICM, and normal groups; (c) the percentage of various types of cells in DCM, ICM, and normal groups; (d) dot plot of the average expression levels of canonical markers across cell-type clusters, where the dot size maps to marker expression ratio, and color gradient correspond to gene expression level from low (green) to high (red).

We first used the scRNA-seq dataset GSE145154 to compare the differences between healthy individuals and HF patients [16]. After a rigorous quality control screening, we retained 126,667 cells, which were classified into 25 clusters using UMAP and cluster analysis. By assessing the presence and abundance of canonical cell signature genes within each cluster, we identified 10 cell clusters (Figure 2(a)) and three sample types (Figure 2(b)). We then used a dot plot to depict the expression levels of signature markers for each of the 10 clusters. The clusters were identified to be T cells (CD3D, CD3E, and CD3G), natural killer (NK) cells (FCGR3A and KLRB1), B cells (CD79A, CD79B, and BANK1), myeloid cells (LYZ, C1QC, and C1QB), endothelial cells (ECs) (VWF, TAGLN, and CLDN5), endocardial cells (LUM and DCN), fibroblast cells (FBs) ((LUM and DCN), pericytes (VWF, TAGLN, and CLDN5), smooth muscle cells (SMCs) (MYH11), cardiomyocyte cells (MYH7 and MYL2) (Figure 2(d)). The 10 cell clusters were then colorized with three colors (green, blue, and red) based on their respective sample source (normal, DCM, and ICM) (Figure 2(b)). As shown in Figure 2(b), the cell distribution in DCM was similar to that in ICM, and the general cellular population proportions did not exhibit meaningful variance. However, the cell composition in the healthy population differed from that in patients with HF, including DCM and ICM. We also calculated the percentage of 10 cell clusters for each sample group and found that HF samples had a significantly lower percentage of ECs, fibroblasts, and myeloid cells and a significantly higher percentage of T cells and NK cells (Figure 2(c)).

Next, we performed a systematic analysis of scRNA-seq (GSE145154) and bulk RNA-seq data (GSE79962, GSE5406, and GSE57338) to further identify the cell types associated with HF. Owing to a significant reduction in the cardiac population of myeloid cells in HF patients, we performed GSEA to explore the enrichment of myeloid cell markers in the bulk datasets. Notably, downregulated genes in DCM (*Supplementary 1*) and ICM (*Supplementary 1*) displayed a prominent enrichment of marker genes from myeloid cells.

### 3.2. Analysis of Differential Gene Expression in the Single-Cell Expression Profile of Myeloid Cells

**Figure 3:**
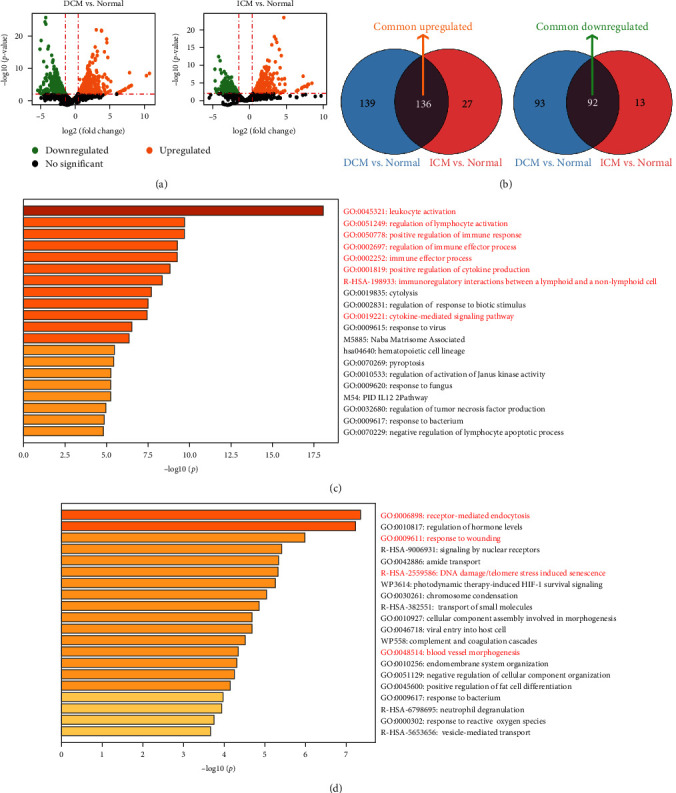
Differential expression analysis of myeloid cells: (a) volcano plot exhibiting the differentially expressed genes among myeloid cells in heart failure compared to the normal (left: DCM vs. normal; right: ICM vs. normal). Orange and green points indicated upregulated and downregulated genes, respectively; (b) Venn diagrams of intersections among genes significantly upregulated (left) and downregulated (right); (c, d) the enriched analysis for common upregulated DEGs (c) and common downregulated DEGs (d) via the Metascape online website. The pathways marked with red were those related with possible pathomechanism of heart failure.

We investigated the dysregulation mechanism underlying HF by conducting differential gene expression analysis to identify and compare dysregulated genes in myeloid cells between DCM/ICM and normal controls. Significantly differentially expressed 275 up- and 185 downregulated genes were identified in DCM (Figure 3(a)). In ICM, 163 and 105 genes were significantly differentially up- and downregulated, respectively (Figure 3(a)). Furthermore, a total of 136 intersection genes were identified as upregulated, with 92 as downregulated among the differentially expressed genes in both DCM and ICM (Figure 3(b)). These genes were associated with common changes in HF due to DCM and ICM (*Supplementary 2*).

To reveal the molecular mechanism involved in HF, we used Metascape to conduct a functional enrichment analysis of common DEGs in myeloid cells. Our analysis revealed that 136 upregulated genes were significantly enriched in inflammatory response-related pathways such as leukocyte activation, regulation of lymphocyte activation, positive regulation of immune response, regulation of immune effector response, immune effect process, positive regulation of cytokine production, immunoregulatory interactions between lymphoid and nonlymphoid cells, and cytokine-mediated signaling pathways (Figure 3(c)). In contrast, 92 downregulated genes displayed a marked enrichment in damage repair-related pathways such as receptor-mediated endocytosis, response to wounding, DNA damage/telomere stress-induced senescence, and blood vessel morphogenesis (Figure 3(d)).

### 3.3. Characterization of the Role of Signaling Pathways and Key Factors in Myeloid Cell Interactions

**Figure 4:**
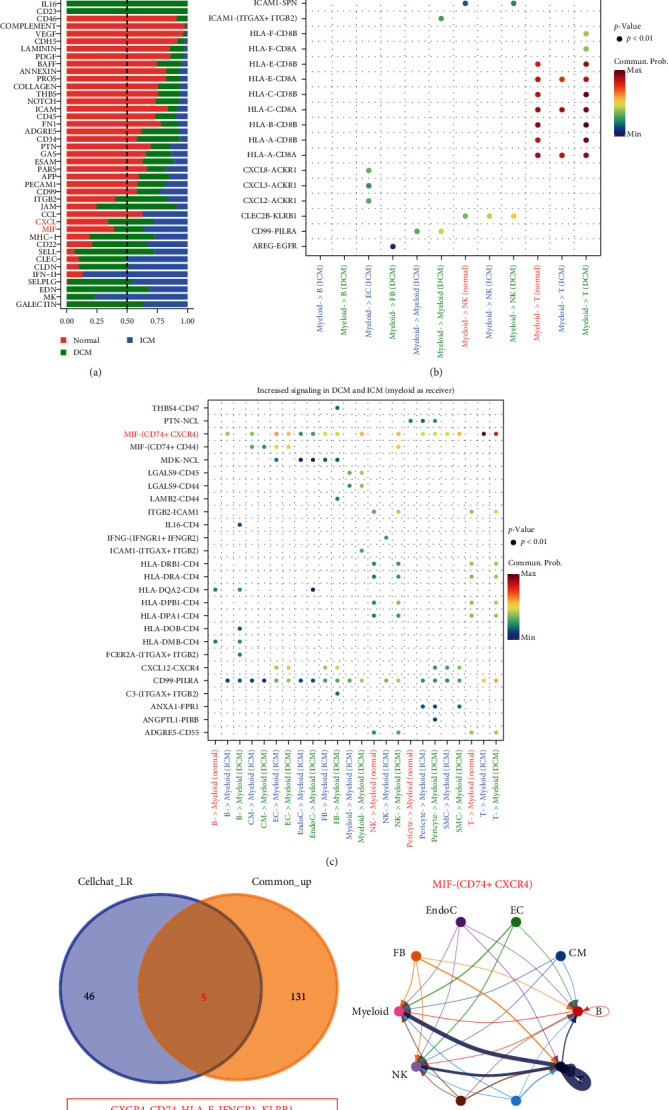
Cell–cell communication signals participated by myeloid cells in HF: (a) bar plot illustrating significant signaling pathways in DCM, ICM, and normal groups. The overall flow of information in a signaling network is accomplished by aggregating all potential communication probabilities within the network; (b, c) comparative analysis of the significantly increased receptor–ligand pairs in ICM and DCM with myeloid cells as senders and receivers; (d) Venn diagrams of intersections between significantly co-upregulated genes in myeloid cells of DCM and ICM (Common_up) and receptor and ligands identified in Cellchat (Cellchat_LR); (e) circle plot for the inferred MIF-(CD74 + CXCR4) signaling network. The arrows indicate that the signal is transmitted from the sender to the receiver, while the thickness of the lines reflects the strength of their interaction.

To predict cell–cell interactions among myeloid cells and other cells, we linked these cell populations based on expression levels and interactions of ligands with their respective receptors. We used the Cellchat R package to deduce cell-specific signal transmission and created Cellchat objects for three groups (normal, DCM, and ICM) to identify significant signals in each group. Signal integration analysis yielded 58 pathways with significant interactions (Figure 4(a)).

Using myeloid cells as signal senders, we found that three CXCL-based interactions, CXCL8-ACKR1, CXCL3-ACKR1, and CXCL2-ACKR1, were enhanced in ICM with respect to the communication between myeloid and ECs (Figure 4(b)). Signals that were significantly enhanced in DCM and ICM with myeloid cells as receivers, including MIF, IL16, MHC-II, CXCL, and CD99 (Figure 4(c)). Surprisingly, we found that the MIF signal was identified as either a receiver or sender of the signal. In addition, we observed significant differences between immune and myeloid cells in terms of the interaction between MHC-II (HLA-DR, HLA-DP, HLA-DO, HLA-DQ, and HLA-DM) and CD4. Furthermore, myeloid cells were found to interact with stromal cells (ECs, SMCs, and fibroblasts) via CXCR4 and CXCL12. In addition, the interaction of the LGALS9-CD45/CD44 pair was strengthened in ICM and DCM but not in controls. We intersected the coupregulated genes in the myeloid cells of patients with DCM and ICM with the recipient ligands identified by CellChat. Five molecules, CXCR4, CD74, HLA-F, IFNGR1, and KLRB1, were found to be significantly upregulated (Figure 4(d)). Among these molecules, CD74 and CXCR4 synergistically work to form a MIF receptor complex. The interaction network of the above signaling pathway (MIF) is depicted in Figure 4(e), which shows that all other cells, especially T cells, can interact with myeloid cells via MIF-CXCR4 + CD74 molecules.

### 3.4. Evaluation of the Diagnostic Performance of CXCR4 and CD74

**Figure 5:**
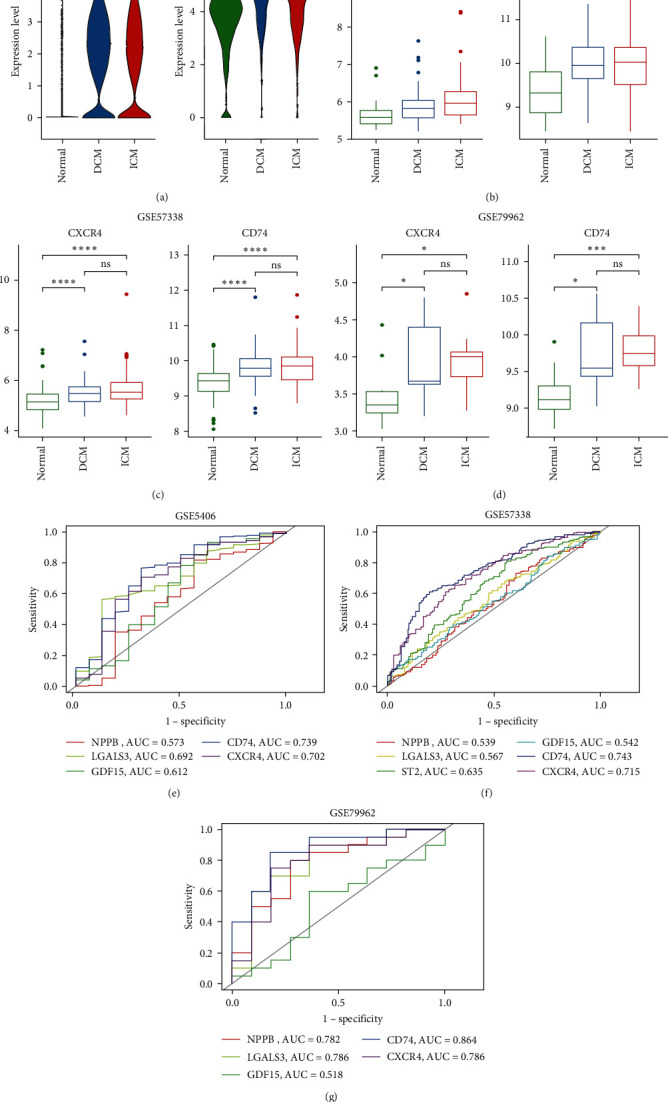
The differential expression of CXCR4 and CD74: (a) violin diagrams display changes in CXCR4 and CD74 expression levels in myeloid cells between DCM/ICM and normal samples; (b–d) box plots of differential expression levels of CXCR4 and CD74 between DCM/ICM and normal samples using bulk RNA-seq dataset of GSE5406, GSE79962, and GSE57338 ( ^*∗*^p < 0.05,  ^*∗∗*^p < 0.01,  ^*∗∗∗*^p < 0.001; ns, not significant); (e–g) ROC curves and AUC values of the five gene markers were delineated and calculated using the datasets GSE5406 (e), GSE57338 (f), and GSE79962 (g).

To investigate the modulatory impact of MIF signaling on HF progression, we uploaded the genes of the CXCR4 + CD74 pair and MIF to the Metascape platform to analyze BPs using GO. The outcomes indicated that MIF signaling molecules were primarily engaged in the regulation of chemotaxis and regulated by NF-*κ*B1 and RELA (*Supplementary 3*). The scRNA-seq data indicated that myeloid cells derived from DCM and ICM exhibited significantly elevated expression levels of CXCR4 and CD74 (Figure 5(a)). In addition, we validated this result in the bulk dataset containing DCM, ICM, and normal samples. Compared with the normal group, we observed consistent upregulation of CXCR4 and CD74 in both DCM and ICM, whereas no significant differences were detected between ICM and DCM (Figure 5(b)–5(d)).

To evaluate the values of CD74 and CXCR4 as predictive biomarkers for HF, we constructed ROC curves and computed AUC values (Figure 5(e)–5(g)). The AUC for CD74 and CXCR4 were 0.739 and 0.702 in GSE5406 (Figure 5(e)), 0.743 and 0.715 in GSE57338 (Figure 5(f)), and 0.864 and 0.786 in GSE79962 (Figure 5(g)), respectively. In addition, we compared the common biomarkers for HF diagnosis, including NPPB, LGALS3, ST2, and GDF15 [34–37]. We found that CD74 and CXCR4 had higher AUC values compared to these common biomarkers, suggested that CD74 and CXCR4 had potential diagnostic value for HF disease.

To further validate the significance of CD74/CXCR4, we conducted a verification experiment using the GSE141910 dataset, which contained 161 DCM-induced HF and 94 normal samples. Consistent with previous results, CXCR4 and CD74 levels were substantially higher in DCM samples than in normal samples (*Supplementary 1*). In addition, CD74 and CXCR4 demonstrated good predictive performances, with AUC values of 0.838 and 0.701, respectively (*Supplementary 1*). LVEF is a key indicator of cardiac systolic function and reflects HF progression. Spearman's correlation analysis revealed that both CXCR4 and CD74 were negatively correlated with LVEF (*Supplementary 1*), indicating that their high expression in the HF group was associated with a decrease in LVEF. These results further support the hypothesis that CXCR4 and CD74 are strongly associated with HF.

### 3.5. ScRNA-Seq Analysis of Myeloid Cells Subpopulations

**Figure 6:**
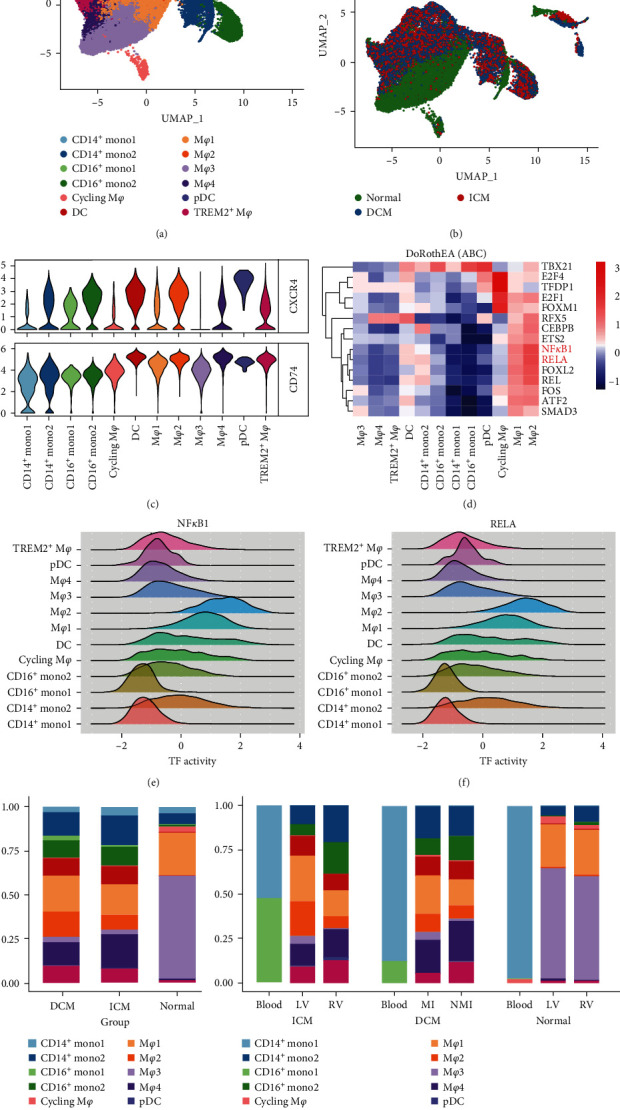
Classification and molecular characterization of myeloid cell subsets: (a) UMAP plots of 12 subclusters of myeloid cells; (b) UMAP plots of myeloid subsets, colored by DCM, ICM, and normal groups; (c) comparison for gene expression of CXCR4 and CD74 in 12 myeloid cell subsets; (d) heatmap of the most variable TFs activity among different myeloid subsets; (e, f) comparison for the distribution of transcription factor activities of NF-*κ*B and RELA in 12 myeloid cell subsets; (g, h) comparison for proportions of different myeloid cell subclusters in three groups (DCM, ICM, and normal) and three sampling regions (blood, LV, and RV), LV: left ventricle. RV: right ventricle, MI: infarcted myocardium, NMI: noninfarcted myocardium.

The major group of immune cells observed in patients with HF are myeloid cells, particularly macrophages. Previous studies have shown that macrophages have diverse subgroups performing specialized functions in response to changes in the immune environment [38, 39]. In order to gain deeper insight into the heterogeneity of the diverse macrophage subgroups in healthy individuals and patients with HF, we investigated the transcriptional changes and heterogeneity of myeloid cells. Using UMAP analysis, we divided myeloid cells into 12 subgroups (Figure 6(a)) and found that myeloid cells of DCM/ICM were distinct from normal samples (Figure 6(b)). We also evaluated the CXCR4 and CD74 expression among different cellular subsets and found that CD74 was highly expressed in all cell subtypes, whereas CXCR4 was highly expressed in all myeloid subsets except M*φ*3, cycling M*φ*, and CD14^+^ mono1 cells (Figure 6(c)). To determine the association between changes in gene expression and intracellular signaling, we evaluated TF activity in myeloid cell subclusters using Dorothea. Results showed that cycling M*φ* had the highest TF activity, followed by M*φ*2 and M*φ*1(Figure 6(d)). In particular, the TFs NF*K*B1 and RELA were activated in M*φ*2 and M*φ*1 (Figure 6(e)–6(f)).

To illustrate the variation in cell subtypes, we calculated the relative proportions of cell subtypes in each group. The constructed histograms showed remarkable changes in the proportion of cell subtypes. The main subtypes of myeloid cells in the normal samples were M*φ*1 and M*φ*3 (Figure 6(g)). On the other hand, there was a noticeable rise in the percentage of M*φ*2 and M*φ*4, dendritic cells, and monocytes (CD14^+^ mono2, CD16^+^ mono2) increased significantly in DCM and ICM, while the proportion of M*φ*3 decreased (Figure 6(g)). In terms of sampling tissue sources, there was a noteworthy increase in the proportion of CD16^+^ monocytes in the blood of individuals with DCM and ICM, while the proportion of M*φ*2 was significantly raised in the left ventricle of DCM and in the area of infarction for ICM (Figure 6(h)).

### 3.6. Functional Analysis of Myeloid Cell Subclusters

**Figure 7:**
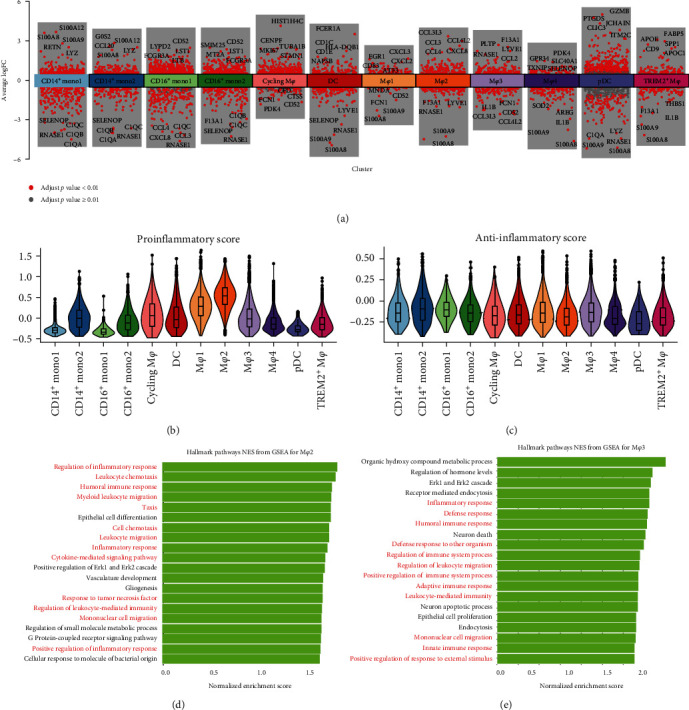
Functional analysis of myeloid cell subsets: (a) differential gene expression analysis of 12 myeloid cell subclusters. The adjusted *p* value < 0.01 is indicated in red, whereas the adjusted *p* value > = 0.01 is indicated in gray; (b, c) violin plot of the proinflammatory score and anti-inflammatory score for all 12 subsets; (d, e) top 20 of GO terms and pathways enriched in upregulated genes of M*φ*2 and M*φ*3.

To investigate the function of each myeloid cell subcluster, we performed differential gene expression analysis for each subcluster (Figure 7(a)). A volcano plot showed that M*φ*2 showed high level of expression for inflammatory chemokines (CXCL2, CXCL3, and CXCL8) and related genes (CCL3, CCL4, CCL3L3, and CCL4L2) (Figure 7(a)). Proinflammatory scores also revealed that M*φ*2 was the primary proinflammatory cluster (Figure 7(b)). Anti-inflammatory scores also revealed that M*φ*3 was the relatively anti-inflammatory cluster (Figure 7(c)). GO analysis of the upregulated genes in M*φ*2 showed enrichment for granulocyte chemotaxis and migration and antigen processing and presentation (*Supplementary 1*). KEGG enrichment analysis revealed that inflammatory pathways enriched in M*φ*2 subsets, such as the NF-kappa B, IL-17, and TNF signaling pathways (*Supplementary Figure S3B*). GSEA further revealed the enrichment of several biological pathways related to the regulation of inflammation and cellular chemotaxis in M*φ*2 (Figure 7(d)). On the other hand, M*φ*3 showed a high level of expression for LYVE1, which is considered a marker of tissue-resident macrophages (Figure 7(a)). GO and KEGG enrichment analysis revealed that M*φ*3 was correlated with endocytosis (*Supplementary 1*). GSEA also revealed the enrichment in M*φ*3 for several biological pathways related to the regulation of inflammation and endocytosis (Figure 7(e)).

### 3.7. The Differentiation Trajectory of Myeloid Cell Subsets

**Figure 8:**
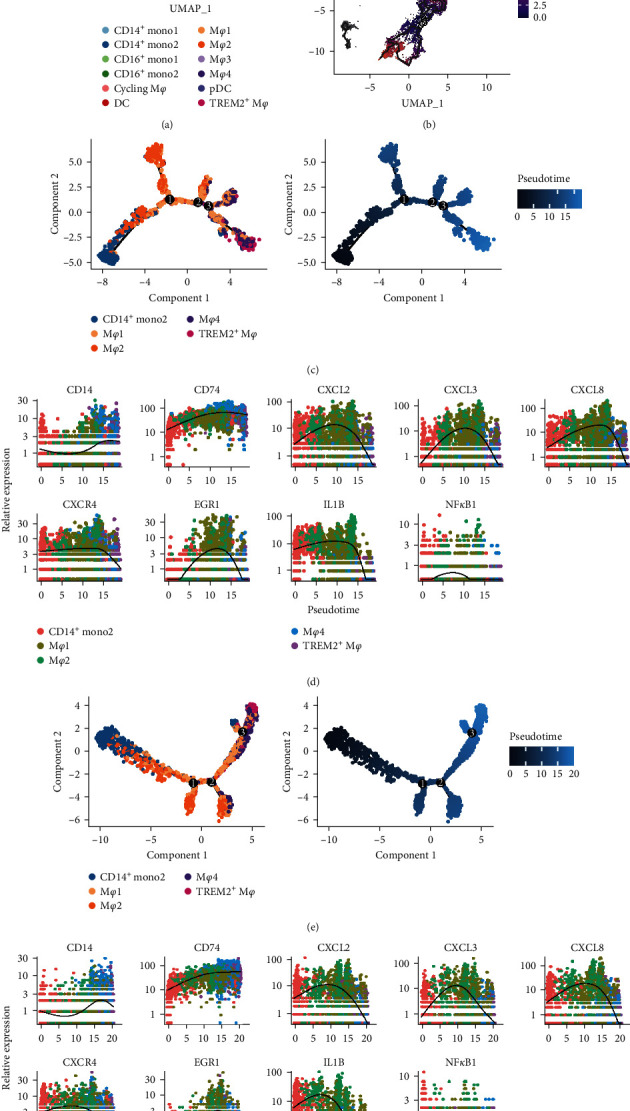
Macrophage phenotype transformation: (a, b) UMAP visualization of cells ordered along trajectories with monocle 3 and labeled by defined myeloid cell subsets (a) or by inferred pseudotime (b); (c, e) cells trajectory analysis in DCM (c) and ICM (e), including the CD14^+^ monocyte subset, M*φ*1 subset, M*φ*2 subset, M*φ*4 subset, and TREM2^+^ M*φ*, colored by identified subset or inferred pseudotime with monocle3; (d, f) pseudotime kinetics of indicated genes from cell subset of DCM (d) and ICM (f).

Finally, we conducted pseudotime analyses to deduce differentiation trajectories of myeloid cell subsets to gain a better understanding of their transitions. Using Monocle3, we constructed a developmental trajectory and superimposed it onto the trajectory cluster defined by Seurat (Figure 8(a)). Cell populations were redistributed in Monocle3, and cell populations in the normal group were clearly separated from those in the HF group using faceted plots (*Supplementary 1*). The M*φ*3 was observed to be clearly separated from monocytes. Moreover, some parts were found in the same branch as M*φ*1 (Figure S4B). Considering the high expression of tissue-resident marker (LYVE1), the M*φ*3 may be a resident-like subset not derived from monocytes. Although M*φ*1 was divided into two trajectories, except for a portion of the trajectory that overlapped with that of M*φ*3, the rest of its trajectory was closely associated with M*φ*4 and TREM2^+^ macrophages (Figure 8(a) and *Supplementary 1*). M*φ*2, which is related to proinflammatory responses, showed the shortest branching distance to monocytes and may thus originate from monocyte differentiation (Figure 8(a) and *Supplementary 1*).

To investigate the relationship between monocytes and macrophages in terms of their differentiation behavior, four macrophage subsets and CD14^+^ monocytes in the DCM and ICM groups were analyzed using Monocle2. In the DCM group, the pseudotime trajectory showed that CD14^+^ monocytes were the starting point, followed by M*φ*2 and M*φ*1, and then M*φ*4 or TREM2^+^ M*φ* (Figure 8(c)). Similar macrophage transformation trajectories were observed in the ICM group (Figure 8(e)). The expression of inflammatory factors displayed a clearly increasing trend, followed by downregulation during macrophage differentiation (Figure 8(f)).

## 4. Discussion

HF, which represents the terminal phase of various cardiac diseases, is frequently linked to poor outcomes and high mortality rates [3, 4]. Earlier studies propose that inflammation is a pivotal factor in the advancement of HF [7, 11]. Advances in single-cell sequencing technologies have facilitated the study of cell-type variety in HF, crosstalk mechanisms between cell types, and particular molecular characteristics of cell differentiating phases. Here, our objective is to analyze the crucial cell types and regulatory mechanisms that underlie HF, with the aim of identifying new diagnostic and therapeutic targets.

We evaluated cell abundance of myeloid cells using scRNA-seq, and then we performed pseudo-bulk differential gene analysis and interaction regulation to gain further insights. Our results showed that the molecules involved in the MIF pathway were significantly upregulated in HF and could potentially serve as biomarkers. Furthermore, our analysis of myeloid cell subtypes at a higher resolution revealed an increased number of proinflammatory macrophages and a decreased number of anti-inflammatory macrophages. This is consistent with previous studies using mouse models of chronic HF [40, 41]. Macrophages are the predominant immune cells in the heart and have an important function in maintaining cardiac homeostasis [9, 12, 42]. Here, we divided myeloid cells into 12 subsets, including 6 subsets of macrophages. Our findings suggest that the drop of anti-inflammatory M*φ*3, which exerts a cardioprotective effect, and the substantial upregulation of proinflammatory M*φ*2 are associated with HF. Our analysis of TF activity also revealed that NF-*κ*B-related TFs were active in M*φ*2, which may be derived from monocytes as evidenced by differentiation trajectory analysis results [43, 44].

**Figure 9:**
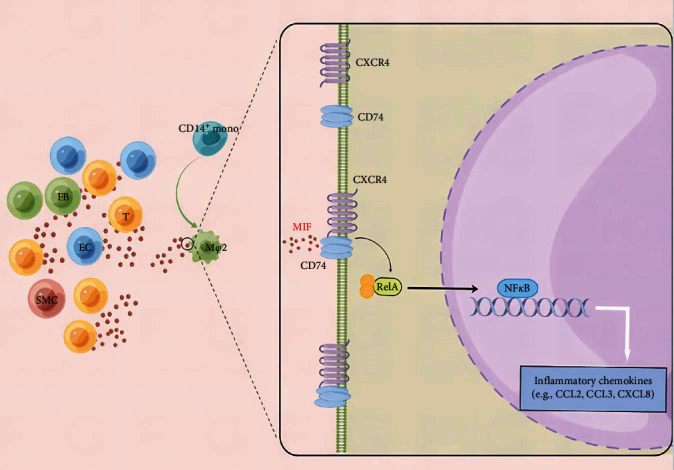
Schematic diagram. MIF-(CD74 + CXCR4) signal transduction cascade mediates the production of inflammatory chemokines in macrophages.

Macrophage migration inhibitory factor (MIF) is an immunomodulatory molecule expressed by various cell types, including eosinophils [45], macrophages [46], epithelial cells [47], ECs [48], and lymphocytes [49]. MIF expression is significantly correlated with HF development [50]. As an MIF receptor, CD74 plays a critical role in the synthesis and trafficking of MHC class II molecules [51]. MIF initiates the inflammatory cascade by forming a ligand–receptor complex with CD74′s extracellular domain, and macrophage MIF initializes the inflammatory cascade [52]. In addition to CD74, CXCR4 is also involved in MIF-induced signal transduction [52, 53]. Previous studies have reported that the CXCR4 and CD74 complex colocalizes at the cell membrane and mediate MIF-specific signal transduction [54]. Several studies indicated that exposing cardiac myofibroblasts to sCD74 and MIF leads to the apoptosis of fibroblasts during scar maturation [55]. Moreover, MIF deficiency inhibited NF-*κ*B-mediated inflammatory responses, resulting in the protection of the heart from serious damage in a mouse model of myocardial ischemia-reperfusion [56]. In summary, the CD74/CXCR4 receptor complex combined with MIF activates NF-*κ*B signaling to produce inflammatory chemokines, which form a proinflammatory macrophage phenotype (Figure 9).

B-type natriuretic peptide levels have recently been used as benchmarks for HF diagnosis [37]. Although new biomarkers such as galectin-3, growth differentiation factor 15 (GDF-15), and soluble suppression of tumorigenicity-2 have been proposed, their reliability remains controversial [34–36]. Sequencing technology is becoming faster and cheaper, facilitating large-scale analysis of multiple histological data to better understand and treat patients with HF. New biomarkers are being explored at different levels, including the epigenetic, proteomic [57], and metabolomic [58] levels, to elucidate the pathogenesis and treatment of HF [6]. In addition, the integration of clinical indicators with genomic studies can guide patient classification and precise treatment better [59]. In our study, we identified upregulated MIF signaling pathways and CD74/CXCR4 receptors in cardiac patients with advanced HF. Through the evaluation of ROC curves and AUC values via external dataset validation (GSE5406, GSE57338, GSE79962, and GSE141910), CD74 and CXCR4 demonstrated robust diagnostic performance in identifying high-risk individuals for HF, showing AUC values surpassing 0.7. Moreover, correlation analysis revealed that the expression of CXCR4 and CD74 was negatively correlated with cardiac contractile function.

In summary, this study confirmed the involvement of myeloid cells in the progression of HF based on an analysis of scRNA-seq and bulk RNA-seq data and identified CXCR4 and CD74 as potential biomarkers of HF based on cellular interactions. Although the effectiveness of these molecules has been validated with single-cell and bulk transcriptome datasets, there is still considerable work required before effective drug development for HF can be achieved. The effectiveness of drugs targeting CXCR4 and CD74 in treating HF needs to be verified through further experimental exploration in follow-up studies.

## 5. Conclusions

In conclusion, our research combined scRNA-seq and bulk RNA-seq techniques to explore HF in depth and revealed key genes. Specifically, we found heterogeneity in functional enrichment, cell differentiation trajectories, and intercellular communication in myeloid cells in HF. In addition, key genes identified by cellular interaction and differential analysis were shown to be of potential diagnostic value. With the present study, we not only expand the understanding of HF-associated macrophages but also provide a new mechanism that may be involved in the regulation of HF progression. We hope that this combined analysis using scRNA-seq and bulk RNA-seq will advance the further development of diagnosis and treatment for HF. Of course, further experiments and clinical practice are needed to confirm the results of this study.

## Figures and Tables

**Table 1:** A summary of the dataset.

Accession	RNA library	Sample size	Source
GSE145154	Single-cell RNA sequencing	Normal:1, DCM:2, ICM:3	Heart and blood
GSE5406	Bulk RNA sequencing	Normal:16, DCM:86, ICM:108	Left ventricle
GSE57338	Bulk RNA sequencing	Normal:136, DCM:82, ICM:95	Left ventricle
GSE79962	Bulk RNA sequencing	Normal:11, DCM:9, ICM:11	Left ventricle
GSE141910	Bulk RNA sequencing	Normal:94, DCM:161	Left ventricle

## Data Availability

The datasets presented in this study can be found in the GEO database (https://www.ncbi.nlm.nih.gov/geo). The names of the repository/repositories and accession number(s) can be found in Section 2.

## Supplementary Materials

Supplementary 1 Figure S1: GSEA analysis of myeloid cell markers; Figure S2: negative correlation between CXCR4 and CD74 with left ventricular ejection fraction; Figure S3: functional enrichment analysis of GO and KEGG in myeloid cell subsets; Figure S4: cell differentiation trajectory inferenced by monocle 3.

Supplementary 2 Table S2: results of DEGs in myeloid cells.

Supplementary 3 Table S1: GO biological process annotation of CXCR4, CD74, and MIF.
