# Supplementary material for: Key Cell Types and Biomarkers in Heart Failure Identified through Analysis of Single-Cell and Bulk RNA Sequencing Data
Source: Mediators Inflamm. 2023 Dec 26;2023:8384882. doi: 10.1155/2023/8384882 (PMC10761229; doi:10.1155/2023/8384882)
Supplement: Supplementary 1 — Figure S1: GSEA analysis of myeloid cell markers; Figure S2: negative correlation between CXCR4 and CD74 with left ventricular ejection fraction; Figure S3: functional enrichment analysis of GO and KEGG in myeloid cell subsets; Figure S4: cell differentiation trajectory inferenced by monocle 3. [file 8384882.f1.docx]

Supplementary Material

Key cell types and novel biomarkers in heart failure identified through analysis of single-cell and bulk RNA sequencing data

Ying Kong^1,2^, Ning Yang^1^, Zhiqing Luo^1^, Ruiting Huang^1^, Quhuan Li ^1,^ ^2^*

*** Correspondence: Quhuan Li ：**[**liqh@scut.edu.cn**](mailto:liqh@scut.edu.cn)


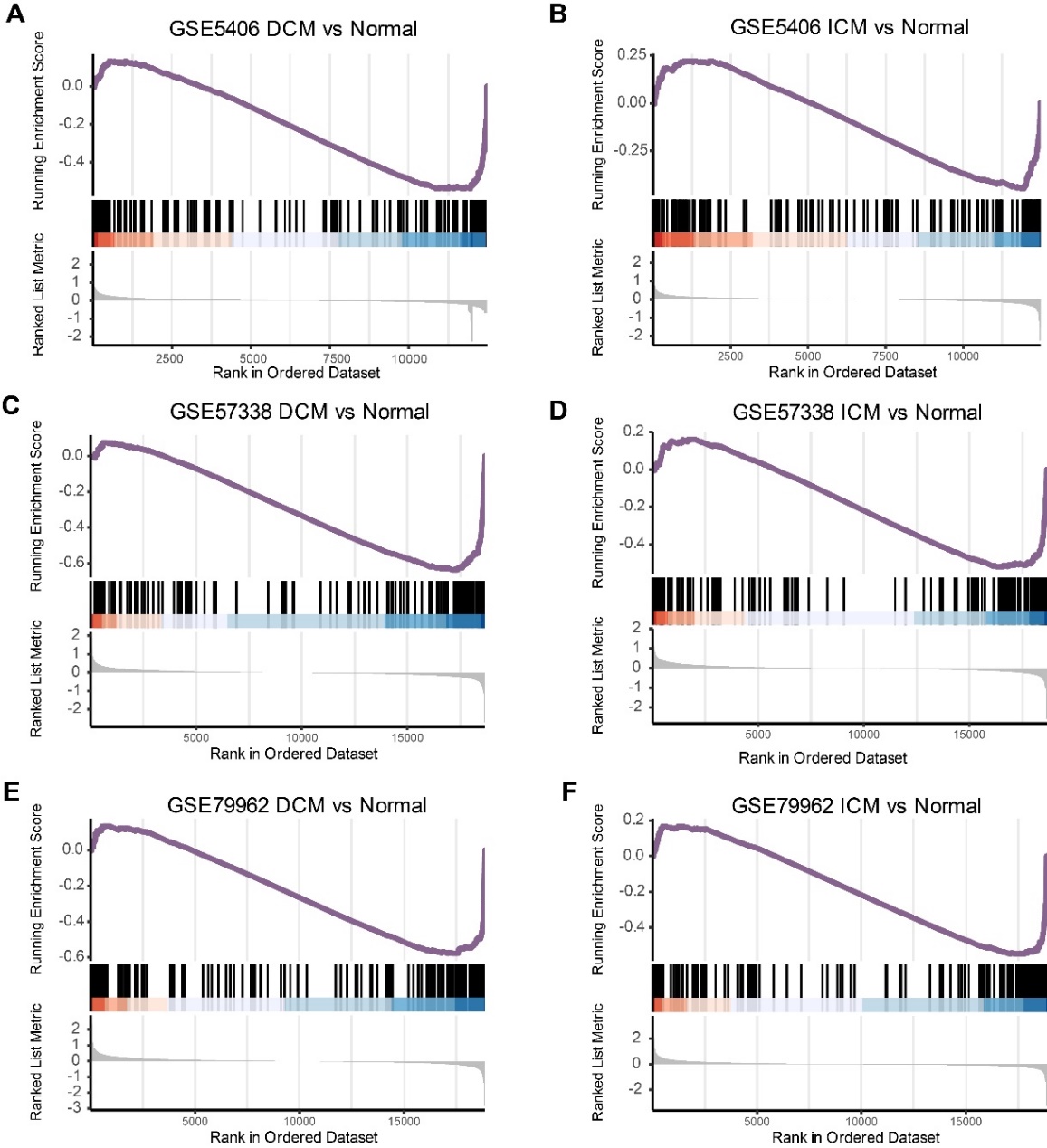


**Figure S1 GSEA analysis of myeloid cell markers.** The marker genes of myeloid cell types enriched the downregulated genes of DCM (A, C, E) or ICM (B, D, F) using datasets of GSE5406(A,B), GSE57338(C, D), GSE79962(E, F).


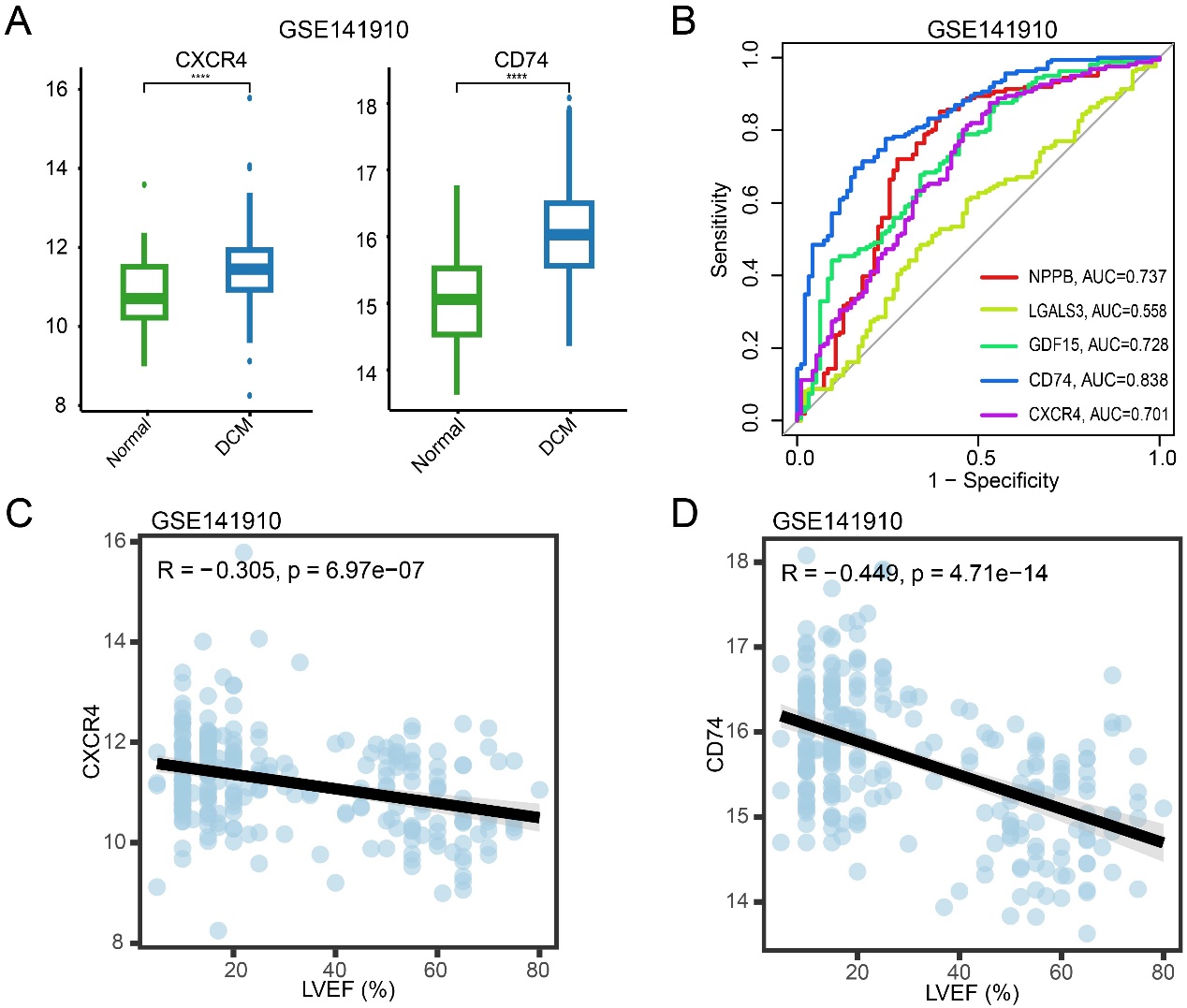


**Figure S2 Correlation between CXCR4/CD74 and left ventricular ejection fraction.** (A) The box plots illustrate the expression levels of CXCR4 and CD74 in dilated cardiomyopathy (DCM) and normal samples from the GSE141910 dataset (****p <0.0001). (B) ROC curves and AUC values of the five gene markers were delineated and calculated using the GSE141910 dataset. (C–D) Scatterplot shows the correlation between CXCR4 (C) and CD74 (D) expression level and LVEF.


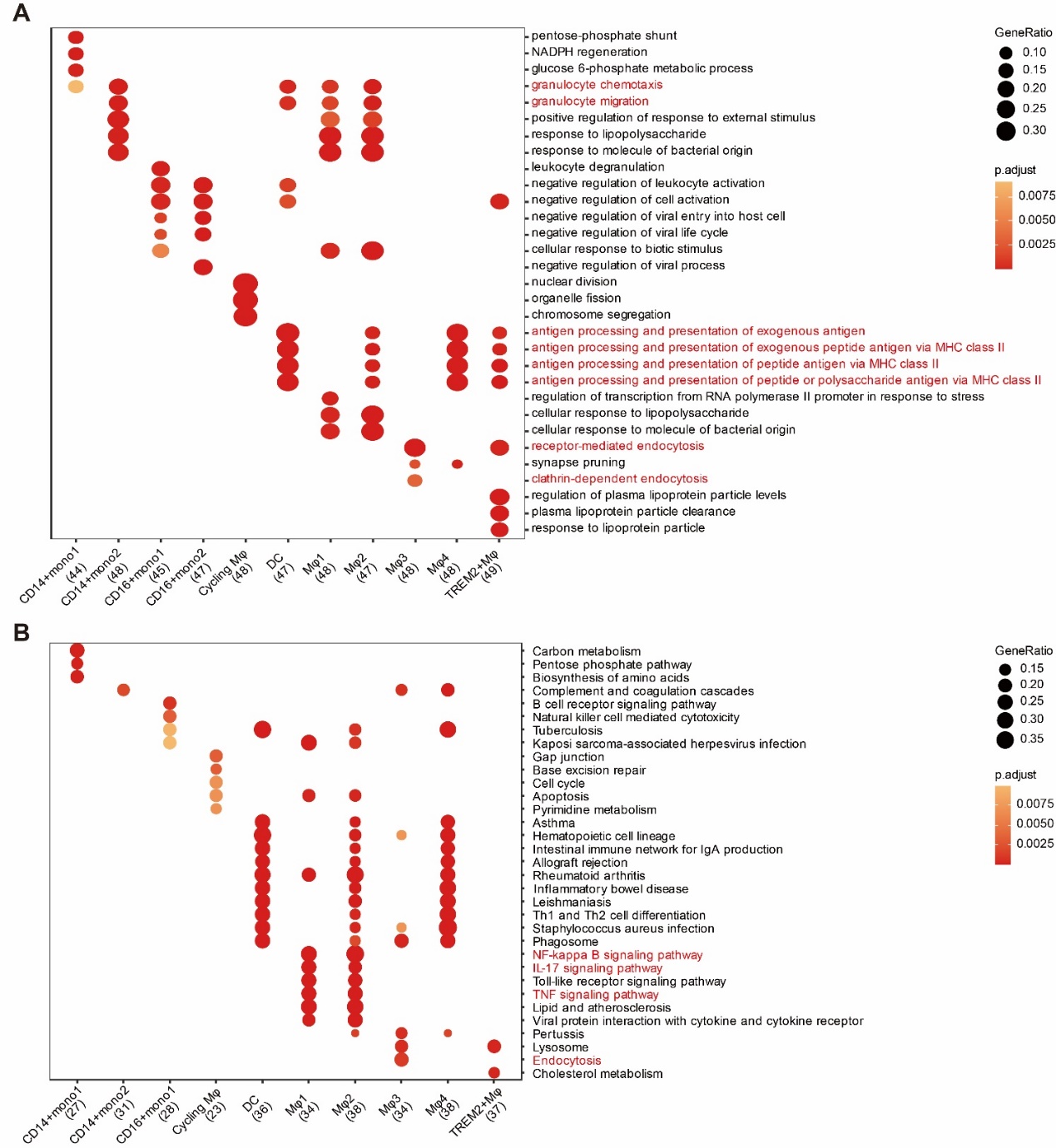


**Figure S3 Functional enrichment analysis of GO and KEGG in myeloid cell subsets.** (A) Dot plot of GO pathway for upregulated genes from each myeloid cell subsets. (B) Dot plot of KEGG pathway for upregulated genes from myeloid cell subsets.

**
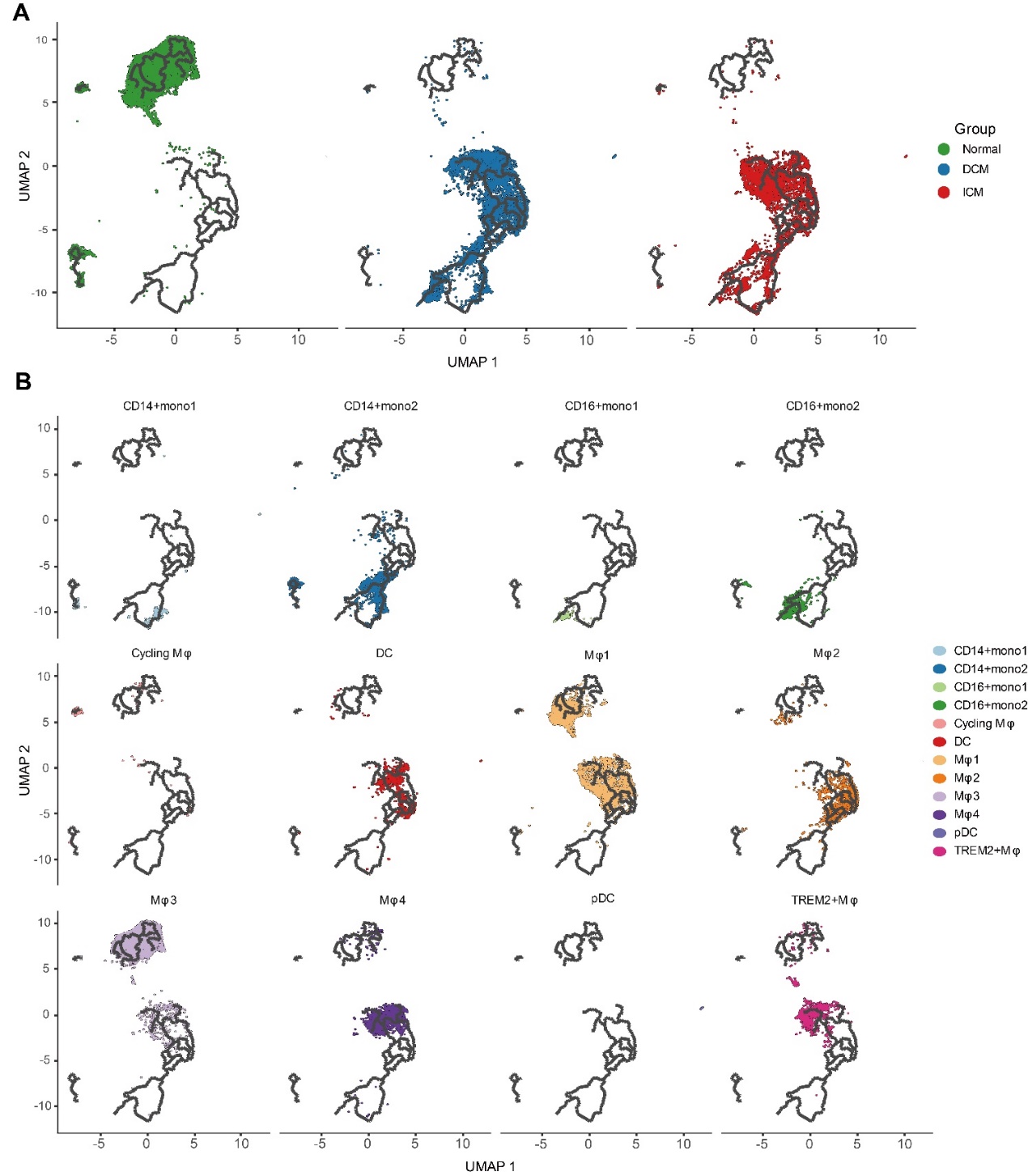
**

**Figure S4 Cell differentiation trajectory inferenced by monocle 3.** (A) The prediction of myeloid cell subsets differentiation trajectories split by groups (Normal, DCM, ICM) with Monocle 3. (B) Visualization of three groups of cardiac macrophage trajectories colored by 12 subsets.
