# Supplementary material for: Key Cell Types and Biomarkers in Heart Failure Identified through Analysis of Single-Cell and Bulk RNA Sequencing Data
Source: Mediators Inflamm. 2023 Dec 26;2023:8384882. doi: 10.1155/2023/8384882 (PMC10761229; doi:10.1155/2023/8384882)
Supplement: Supplementary 2 — Table S2: results of DEGs in myeloid cells. [file 8384882.f2.docx]

Table S2: Results of DEGs in myeloid cells;

| Results of DEGs in myeloid cells | | | | | | | | | | | | |
| --- | --- | --- | --- | --- | --- | --- | --- | --- | --- | --- | --- | --- |
|  | DCM vs Normal | | | | | | ICM vs Normal | | | | | |
| gene | baseMean | log2FC | lfcSE | stat | pvalue | padj | baseMean | log2FC | lfcSE | stat | pvalue | padj |
| ABI3 | 655.8394 | 2.441999 | 0.429736 | 5.68256 | 1.33E-08 | 2.81E-06 | 315.2886 | 2.183519 | 0.509497 | 4.285633 | 1.82E-05 | 0.002179 |
| AC091814.2 | 24.25795 | 8.459096 | 2.308038 | 3.665059 | 0.000247 | 0.010826 | 21.43533 | 9.072628 | 2.302563 | 3.94023 | 8.14E-05 | 0.006991 |
| AC104809.4 | 37.43345 | 9.085166 | 2.227137 | 4.079303 | 4.52E-05 | 0.002699 | 13.17328 | 8.340335 | 2.338178 | 3.567024 | 0.000361 | 0.021214 |
| AFF3 | 65.1671 | 3.686585 | 0.825354 | 4.466673 | 7.94E-06 | 0.000644 | 45.07609 | 3.945049 | 0.811575 | 4.860979 | 1.17E-06 | 0.000241 |
| AIM2 | 25.11113 | 4.449509 | 1.345416 | 3.307163 | 0.000942 | 0.03054 | 15.05593 | 4.490053 | 1.263991 | 3.552281 | 0.000382 | 0.022178 |
| ALOX15B | 47.92624 | 4.97717 | 1.114028 | 4.467723 | 7.91E-06 | 0.000644 | 49.78984 | 5.809062 | 1.166251 | 4.980972 | 6.33E-07 | 0.000148 |
| ANKRD22 | 99.95056 | 4.214437 | 0.790955 | 5.328292 | 9.91E-08 | 1.57E-05 | 52.72675 | 4.076973 | 0.806409 | 5.055717 | 4.29E-07 | 0.000109 |
| BCL11A | 104.5416 | 3.087454 | 0.660064 | 4.677506 | 2.90E-06 | 0.000276 | 53.53308 | 2.906206 | 0.734336 | 3.957597 | 7.57E-05 | 0.006647 |
| C11orf21 | 143.0472 | 2.659677 | 0.595152 | 4.468904 | 7.86E-06 | 0.000644 | 72.32371 | 2.466149 | 0.647637 | 3.807921 | 0.00014 | 0.010295 |
| C12orf75 | 177.2447 | 3.226308 | 0.622088 | 5.186254 | 2.15E-07 | 2.87E-05 | 69.30059 | 2.652734 | 0.637527 | 4.160979 | 3.17E-05 | 0.00322 |
| CACNA2D3 | 123.2108 | 2.573008 | 0.662794 | 3.882061 | 0.000104 | 0.005372 | 68.0394 | 2.536663 | 0.703288 | 3.606864 | 0.00031 | 0.01871 |
| CCL17 | 94.1339 | 3.836451 | 0.759404 | 5.051927 | 4.37E-07 | 5.36E-05 | 44.47617 | 3.543874 | 0.941675 | 3.763372 | 0.000168 | 0.011614 |
| CCL20 | 2030.09 | 1.738738 | 0.411606 | 4.224281 | 2.40E-05 | 0.00163 | 1612.879 | 2.286741 | 0.464987 | 4.917859 | 8.75E-07 | 0.000191 |
| CCL5 | 385.9857 | 2.800451 | 0.532119 | 5.262828 | 1.42E-07 | 2.00E-05 | 291.2271 | 3.21815 | 0.551028 | 5.840261 | 5.21E-09 | 2.82E-06 |
| CCNH | 864.2817 | 1.864625 | 0.450219 | 4.141594 | 3.45E-05 | 0.002195 | 403.0859 | 1.568649 | 0.464533 | 3.376829 | 0.000733 | 0.036203 |
| CD1C | 585.0907 | 3.709396 | 0.50889 | 7.28919 | 3.12E-13 | 1.71E-10 | 385.3486 | 3.903315 | 0.473249 | 8.247903 | 1.61E-16 | 3.93E-13 |
| CD1E | 518.4833 | 5.512075 | 0.605142 | 9.108733 | 8.33E-20 | 1.56E-16 | 166.8551 | 4.64827 | 0.550588 | 8.442374 | 3.11E-17 | 9.48E-14 |
| CD2 | 83.04656 | 2.89084 | 0.710641 | 4.067936 | 4.74E-05 | 0.002791 | 61.03988 | 3.266386 | 0.672812 | 4.854824 | 1.20E-06 | 0.000245 |
| CD247 | 47.86243 | 3.776551 | 1.010706 | 3.736547 | 0.000187 | 0.008683 | 23.70818 | 3.541309 | 0.944622 | 3.748914 | 0.000178 | 0.012033 |
| CD3D | 121.1777 | 2.776724 | 0.653093 | 4.251651 | 2.12E-05 | 0.001466 | 83.24692 | 3.054039 | 0.614992 | 4.965986 | 6.84E-07 | 0.000155 |
| CD48 | 1473.999 | 1.841996 | 0.419692 | 4.388925 | 1.14E-05 | 0.000885 | 757.7316 | 1.70258 | 0.461423 | 3.689847 | 0.000224 | 0.014178 |
| CD52 | 4026.979 | 2.766952 | 0.407195 | 6.795145 | 1.08E-11 | 4.73E-09 | 2360.885 | 2.804645 | 0.633737 | 4.425568 | 9.62E-06 | 0.001303 |
| CD74 | 107918 | 1.305323 | 0.384648 | 3.393548 | 0.00069 | 0.023766 | 8467.885 | 2.804645 | 0.633737 | 4.425568 | 9.62E-06 | 0.001103 |
| CDH23 | 100.5672 | 2.841991 | 0.664989 | 4.273744 | 1.92E-05 | 0.001356 | 61.40297 | 2.93457 | 0.673672 | 4.356084 | 1.32E-05 | 0.001656 |
| CHKA | 318.7076 | 1.992071 | 0.575438 | 3.461832 | 0.000537 | 0.019451 | 168.4101 | 1.893494 | 0.533863 | 3.546776 | 0.00039 | 0.022433 |
| CLEC12A | 992.8225 | 4.03381 | 0.498464 | 8.092474 | 5.85E-16 | 5.48E-13 | 684.2401 | 4.287793 | 0.483759 | 8.863499 | 7.75E-19 | 4.73E-15 |
| CLEC4A | 494.7898 | 1.622533 | 0.451926 | 3.590265 | 0.00033 | 0.013763 | 299.9032 | 1.75533 | 0.458146 | 3.831379 | 0.000127 | 0.009835 |
| CLEC4E | 1110.166 | 2.626207 | 0.491114 | 5.347444 | 8.92E-08 | 1.45E-05 | 702.2435 | 2.78253 | 0.443558 | 6.273206 | 3.54E-10 | 3.08E-07 |
| CLEC4F | 33.96157 | 8.945967 | 2.2806 | 3.922638 | 8.76E-05 | 0.004616 | 17.39245 | 8.746575 | 2.246483 | 3.893452 | 9.88E-05 | 0.008089 |
| CLEC9A | 232.8645 | 2.881601 | 0.616878 | 4.671269 | 2.99E-06 | 0.000281 | 83.73588 | 2.175032 | 0.643697 | 3.37897 | 0.000728 | 0.036203 |
| CLIC3 | 102.4262 | 4.360262 | 0.78825 | 5.531576 | 3.17E-08 | 6.04E-06 | 54.07851 | 4.221845 | 0.753682 | 5.601624 | 2.12E-08 | 9.59E-06 |
| COL9A2 | 66.87365 | 2.860702 | 0.799746 | 3.577014 | 0.000348 | 0.014165 | 46.50208 | 3.152175 | 0.740289 | 4.258032 | 2.06E-05 | 0.002395 |
| CTC-428H11.2 | 81.34037 | 2.424866 | 0.731234 | 3.316127 | 0.000913 | 0.029749 | 57.19823 | 2.740499 | 0.691577 | 3.962683 | 7.41E-05 | 0.006597 |
| CTD-3014M21.1 | 595.1404 | 2.720458 | 0.435059 | 6.253079 | 4.02E-10 | 1.26E-07 | 353.8927 | 2.783407 | 0.543018 | 5.125807 | 2.96E-07 | 8.40E-05 |
| CTD-3116E22.8 | 90.09616 | 4.289158 | 0.882339 | 4.861122 | 1.17E-06 | 0.000126 | 32.55944 | 3.609363 | 0.912053 | 3.957406 | 7.58E-05 | 0.006647 |
| CTSW | 154.9006 | 3.945439 | 0.6539 | 6.0337 | 1.60E-09 | 4.57E-07 | 66.63852 | 3.504532 | 0.695737 | 5.037152 | 4.73E-07 | 0.000115 |
| CXCR3 | 39.63802 | 5.119887 | 1.203386 | 4.254567 | 2.09E-05 | 0.001454 | 22.38095 | 5.071964 | 1.19811 | 4.233305 | 2.30E-05 | 0.002611 |
| CXCR4 | 8454.59 | 3.30098 | 0.422211 | 7.818324 | 5.35E-15 | 4.39E-12 | 4248.859 | 3.098034 | 0.489661 | 6.32689 | 2.50E-10 | 2.35E-07 |
| CYSLTR1 | 242.8286 | 2.710398 | 0.508921 | 5.325778 | 1.01E-07 | 1.57E-05 | 126.08 | 2.567374 | 0.524111 | 4.898531 | 9.66E-07 | 0.000203 |
| DEPTOR | 217.2369 | 2.405995 | 0.657206 | 3.660947 | 0.000251 | 0.01092 | 100.104 | 2.08428 | 0.602629 | 3.458645 | 0.000543 | 0.028661 |
| DHRS9 | 276.8725 | 3.396761 | 0.605091 | 5.613638 | 1.98E-08 | 3.96E-06 | 135.0436 | 3.151791 | 0.61265 | 5.144519 | 2.68E-07 | 7.79E-05 |
| DNAAF1 | 211.5802 | 9.139887 | 1.529027 | 5.977582 | 2.26E-09 | 5.94E-07 | 84.00637 | 8.572527 | 1.622486 | 5.283576 | 1.27E-07 | 4.08E-05 |
| EVI2B | 2375.749 | 1.672983 | 0.387337 | 4.319196 | 1.57E-05 | 0.001142 | 1213.402 | 1.52894 | 0.431867 | 3.5403 | 0.0004 | 0.022883 |
| FAM117B | 190.9839 | 2.368515 | 0.535967 | 4.419144 | 9.91E-06 | 0.000774 | 104.7237 | 2.311465 | 0.574219 | 4.025409 | 5.69E-05 | 0.005255 |
| FAM26F | 1971.054 | 2.63459 | 0.391291 | 6.733079 | 1.66E-11 | 6.81E-09 | 980.5106 | 2.425595 | 0.415563 | 5.836886 | 5.32E-09 | 2.82E-06 |
| FCER1A | 2031.992 | 2.78642 | 0.44351 | 6.282661 | 3.33E-10 | 1.09E-07 | 1130.49 | 2.745636 | 0.494013 | 5.557819 | 2.73E-08 | 1.11E-05 |
| FCN1 | 3251.081 | 1.56142 | 0.399859 | 3.904923 | 9.43E-05 | 0.004928 | 2642.196 | 2.156909 | 0.593819 | 3.632267 | 0.000281 | 0.017304 |
| FGFBP2 | 77.57779 | 4.067023 | 0.810514 | 5.01783 | 5.23E-07 | 6.07E-05 | 38.35593 | 3.838318 | 0.811786 | 4.728236 | 2.26E-06 | 0.000432 |
| FGL2 | 3844.773 | 1.3672 | 0.382885 | 3.570789 | 0.000356 | 0.014416 | 2148.761 | 1.383015 | 0.41628 | 3.322316 | 0.000893 | 0.042034 |
| FKBP5 | 1735.022 | 1.850156 | 0.402864 | 4.592509 | 4.38E-06 | 0.000394 | 1159.873 | 2.122525 | 0.496243 | 4.277188 | 1.89E-05 | 0.002219 |
| FLT3 | 91.77323 | 2.614198 | 0.717021 | 3.645918 | 0.000266 | 0.01151 | 59.71593 | 2.805203 | 0.746666 | 3.756969 | 0.000172 | 0.011717 |
| GABPB1-AS1 | 377.1279 | 1.524844 | 0.455326 | 3.348907 | 0.000811 | 0.027116 | 258.7338 | 1.857286 | 0.465269 | 3.991857 | 6.56E-05 | 0.005879 |
| GAPT | 526.1713 | 3.296772 | 0.468935 | 7.030339 | 2.06E-12 | 9.32E-10 | 241.3483 | 2.957444 | 0.481661 | 6.1401 | 8.25E-10 | 5.92E-07 |
| GNLY | 317.8363 | 3.028171 | 0.523005 | 5.789946 | 7.04E-09 | 1.65E-06 | 200.6023 | 3.166926 | 0.542124 | 5.841701 | 5.17E-09 | 2.82E-06 |
| GZMA | 199.2265 | 3.142696 | 0.625763 | 5.022181 | 5.11E-07 | 5.99E-05 | 97.39057 | 2.906278 | 0.694346 | 4.185631 | 2.84E-05 | 0.002964 |
| GZMB | 494.5252 | 4.018802 | 0.520033 | 7.727977 | 1.09E-14 | 7.55E-12 | 187.7866 | 3.397203 | 0.563347 | 6.030388 | 1.64E-09 | 1.05E-06 |
| GZMH | 133.959 | 3.617426 | 0.657308 | 5.503399 | 3.73E-08 | 6.89E-06 | 78.53609 | 3.647381 | 0.660909 | 5.518735 | 3.41E-08 | 1.34E-05 |
| HCP5 | 145.8014 | 2.833814 | 0.587213 | 4.825874 | 1.39E-06 | 0.000147 | 70.44661 | 2.587967 | 0.691326 | 3.743485 | 0.000181 | 0.012134 |
| HERPUD1 | 8697.453 | 2.221311 | 0.433667 | 5.122152 | 3.02E-07 | 3.85E-05 | 3046.283 | 1.463503 | 0.422884 | 3.460763 | 0.000539 | 0.028661 |
| HLA-F | 682.5073 | 2.180238 | 0.425896 | 5.119182 | 3.07E-07 | 3.87E-05 | 301.5569 | 1.792427 | 0.455265 | 3.937111 | 8.25E-05 | 0.007033 |
| ICAM3 | 317.8529 | 2.204995 | 0.474251 | 4.649428 | 3.33E-06 | 0.000309 | 196.0914 | 2.334964 | 0.505887 | 4.615587 | 3.92E-06 | 0.000683 |
| IDO1 | 54.46787 | 6.173793 | 1.273543 | 4.847729 | 1.25E-06 | 0.000133 | 25.55162 | 5.87375 | 1.390218 | 4.225057 | 2.39E-05 | 0.002611 |
| IFI44L | 263.1607 | 1.889577 | 0.502544 | 3.760024 | 0.00017 | 0.008168 | 196.4789 | 2.333645 | 0.515915 | 4.523312 | 6.09E-06 | 0.001003 |
| IFNGR1 | 2192.687 | 2.322064 | 0.471725 | 4.922498 | 8.54E-07 | 9.35E-05 | 1257.095 | 2.338658 | 0.458334 | 5.102515 | 3.35E-07 | 9.08E-05 |
| IL23A | 161.3272 | 3.43772 | 0.697406 | 4.929295 | 8.25E-07 | 9.18E-05 | 83.3369 | 3.286182 | 0.812877 | 4.042654 | 5.28E-05 | 0.004963 |
| IL3RA | 117.1757 | 3.166854 | 0.691707 | 4.578318 | 4.69E-06 | 0.000413 | 40.77935 | 2.402325 | 0.7308 | 3.287255 | 0.001012 | 0.046911 |
| ISG20 | 641.5958 | 2.334048 | 0.439292 | 5.313202 | 1.08E-07 | 1.64E-05 | 359.1822 | 2.313711 | 0.495402 | 4.670371 | 3.01E-06 | 0.000547 |
| JAML | 1328.301 | 2.17462 | 0.417046 | 5.21434 | 1.84E-07 | 2.52E-05 | 808.6932 | 2.287913 | 0.423626 | 5.400779 | 6.64E-08 | 2.25E-05 |
| KCNJ2 | 145.1634 | 2.377926 | 0.577516 | 4.117507 | 3.83E-05 | 0.002349 | 69.29917 | 2.114069 | 0.602168 | 3.510763 | 0.000447 | 0.024881 |
| KLRB1 | 80.18059 | 2.9512 | 0.746572 | 3.953001 | 7.72E-05 | 0.004168 | 60.3994 | 3.364648 | 0.795418 | 4.230039 | 2.34E-05 | 0.002611 |
| KLRD1 | 73.95308 | 2.317219 | 0.726678 | 3.188784 | 0.001429 | 0.041821 | 75.25109 | 3.201029 | 0.707234 | 4.526122 | 6.01E-06 | 0.001003 |
| LILRA4 | 48.038 | 9.445913 | 2.21399 | 4.266467 | 1.99E-05 | 0.001394 | 13.91326 | 8.441315 | 2.273114 | 3.713546 | 0.000204 | 0.013257 |
| LIMD2 | 1186.664 | 2.70546 | 0.399385 | 6.774064 | 1.25E-11 | 5.30E-09 | 591.3241 | 2.496328 | 0.458284 | 5.447126 | 5.12E-08 | 1.84E-05 |
| LINC01970 | 48.00527 | 3.112905 | 0.888006 | 3.505499 | 0.000456 | 0.017438 | 33.67062 | 3.410647 | 0.802107 | 4.252108 | 2.12E-05 | 0.002436 |
| LINC02207 | 202.8429 | 2.115619 | 0.522533 | 4.048773 | 5.15E-05 | 0.002982 | 115.0149 | 2.12553 | 0.549195 | 3.870266 | 0.000109 | 0.00878 |
| LSP1 | 3431.377 | 2.504789 | 0.424099 | 5.906146 | 3.50E-09 | 9.01E-07 | 1154.467 | 1.685619 | 0.420993 | 4.003914 | 6.23E-05 | 0.00567 |
| LTB | 387.522 | 2.606037 | 0.464509 | 5.610305 | 2.02E-08 | 3.96E-06 | 146.9115 | 1.97864 | 0.521585 | 3.793516 | 0.000149 | 0.010531 |
| LY6E | 1315.971 | 1.788777 | 0.397714 | 4.497645 | 6.87E-06 | 0.000574 | 800.2157 | 1.917108 | 0.515901 | 3.716036 | 0.000202 | 0.013198 |
| LYPD2 | 248.5648 | 11.81667 | 2.10277 | 5.619574 | 1.91E-08 | 3.95E-06 | 38.26103 | 9.883117 | 2.260789 | 4.371534 | 1.23E-05 | 0.001584 |
| LYPD3 | 60.37936 | 2.572411 | 0.799916 | 3.21585 | 0.001301 | 0.038881 | 52.2368 | 3.21222 | 0.716159 | 4.485347 | 7.28E-06 | 0.001104 |
| MT2A | 3191.01 | 2.465482 | 0.382187 | 6.450991 | 1.11E-10 | 4.05E-08 | 2999.109 | 3.223674 | 0.640358 | 5.034178 | 4.80E-07 | 0.000115 |
| MTMR11 | 108.2686 | 2.601002 | 0.638224 | 4.075371 | 4.59E-05 | 0.002728 | 65.71267 | 2.686313 | 0.663799 | 4.046875 | 5.19E-05 | 0.004945 |
| MYCL | 85.19911 | 2.584482 | 0.696659 | 3.709824 | 0.000207 | 0.009459 | 43.57797 | 2.415766 | 0.712302 | 3.391491 | 0.000695 | 0.034885 |
| NAPSB | 769.9053 | 3.335225 | 0.428859 | 7.776969 | 7.43E-15 | 5.73E-12 | 426.1592 | 3.274365 | 0.471528 | 6.944157 | 3.81E-12 | 5.16E-09 |
| NCF1 | 286.172 | 2.805309 | 0.584713 | 4.797755 | 1.60E-06 | 0.000167 | 116.5007 | 2.290859 | 0.642687 | 3.564501 | 0.000365 | 0.021271 |
| NKG7 | 599.4944 | 1.626622 | 0.453647 | 3.585659 | 0.000336 | 0.01392 | 423.6122 | 1.999429 | 0.522087 | 3.829688 | 0.000128 | 0.009841 |
| NLRP3 | 4589.466 | 3.200222 | 0.421105 | 7.599579 | 2.97E-14 | 1.95E-11 | 1515.114 | 2.360916 | 0.561242 | 4.206596 | 2.59E-05 | 0.00278 |
| NMUR1 | 67.55518 | 5.477666 | 1.021729 | 5.361172 | 8.27E-08 | 1.36E-05 | 37.55356 | 5.392473 | 1.098546 | 4.908736 | 9.17E-07 | 0.000196 |
| NUDT16 | 890.6181 | 1.913695 | 0.411706 | 4.648206 | 3.35E-06 | 0.000309 | 559.7687 | 2.087244 | 0.503585 | 4.144774 | 3.40E-05 | 0.003428 |
| P2RY13 | 615.6629 | 3.696962 | 0.43647 | 8.470139 | 2.45E-17 | 2.92E-14 | 315.6079 | 3.522167 | 0.512152 | 6.877195 | 6.10E-12 | 6.77E-09 |
| PDK4 | 5439.675 | 3.862466 | 0.394303 | 9.795677 | 1.18E-22 | 3.86E-19 | 1321.936 | 2.56429 | 0.485376 | 5.283096 | 1.27E-07 | 4.08E-05 |
| PIK3IP1 | 264.9005 | 1.82412 | 0.524913 | 3.475093 | 0.000511 | 0.018617 | 164.8448 | 1.986779 | 0.505453 | 3.93069 | 8.47E-05 | 0.007124 |
| PKP2 | 356.9237 | 2.831801 | 0.514959 | 5.499085 | 3.82E-08 | 6.96E-06 | 135.0907 | 2.197842 | 0.589463 | 3.728547 | 0.000193 | 0.012764 |
| PLD4 | 494.5724 | 5.543851 | 0.62803 | 8.827367 | 1.07E-18 | 1.56E-15 | 200.6843 | 5.020851 | 0.637401 | 7.877071 | 3.35E-15 | 6.81E-12 |
| PMAIP1 | 832.3683 | 2.574862 | 0.411617 | 6.255482 | 3.96E-10 | 1.26E-07 | 693.2322 | 3.149195 | 0.697143 | 4.517287 | 6.26E-06 | 0.001018 |
| PRAM1 | 252.2951 | 2.084819 | 0.496253 | 4.201119 | 2.66E-05 | 0.001788 | 156.931 | 2.230269 | 0.557707 | 3.998998 | 6.36E-05 | 0.005746 |
| PRF1 | 101.2058 | 3.08886 | 0.736473 | 4.194127 | 2.74E-05 | 0.001816 | 47.13796 | 2.774425 | 0.683065 | 4.06173 | 4.87E-05 | 0.004677 |
| PSMB9 | 1273.068 | 2.059135 | 0.396095 | 5.198589 | 2.01E-07 | 2.72E-05 | 591.9136 | 1.754839 | 0.429115 | 4.089433 | 4.32E-05 | 0.004253 |
| PTGDS | 216.3612 | 5.15378 | 0.639538 | 8.058605 | 7.72E-16 | 6.75E-13 | 91.59383 | 4.678859 | 0.829439 | 5.640991 | 1.69E-08 | 7.93E-06 |
| RNF144B | 1377.689 | 2.438668 | 0.41966 | 5.81106 | 6.21E-09 | 1.48E-06 | 713.1992 | 2.295104 | 0.51488 | 4.457552 | 8.29E-06 | 0.001204 |
| RNU12 | 101.3283 | 2.726177 | 0.655191 | 4.160887 | 3.17E-05 | 0.00207 | 66.79569 | 2.951195 | 0.660379 | 4.468941 | 7.86E-06 | 0.001167 |
| RP11-1143G9.4 | 1768.956 | 4.701862 | 0.484182 | 9.710929 | 2.71E-22 | 5.92E-19 | 2035.083 | 5.695153 | 0.560836 | 10.15475 | 3.16E-24 | 3.85E-20 |
| RP11-153M7.3 | 102.1657 | 2.406903 | 0.665679 | 3.61571 | 0.0003 | 0.01264 | 54.56056 | 2.30809 | 0.678983 | 3.399336 | 0.000675 | 0.034181 |
| RP11-362F19.1 | 59.35797 | 3.231702 | 1.010107 | 3.199367 | 0.001377 | 0.040619 | 45.73824 | 3.652379 | 0.970863 | 3.761992 | 0.000169 | 0.011614 |
| RP11-365O16.3 | 92.74249 | 4.213049 | 0.762045 | 5.528608 | 3.23E-08 | 6.05E-06 | 100.9707 | 5.14368 | 0.8276 | 6.215175 | 5.13E-10 | 3.91E-07 |
| RP11-404O13.5 | 124.3811 | 2.084613 | 0.608746 | 3.424441 | 0.000616 | 0.021676 | 131.1646 | 3.03469 | 0.552012 | 5.497504 | 3.85E-08 | 1.47E-05 |
| RP11-467L13.7 | 610.9694 | 1.913963 | 0.4417 | 4.333171 | 1.47E-05 | 0.001078 | 350.7504 | 1.949525 | 0.49782 | 3.91612 | 9.00E-05 | 0.007465 |
| RP11-596C23.2 | 133.7216 | 1.88724 | 0.587415 | 3.212788 | 0.001315 | 0.039032 | 87.75065 | 2.134324 | 0.615662 | 3.466715 | 0.000527 | 0.028556 |
| RP11-701P16.2 | 225.7755 | 2.795171 | 0.555636 | 5.030582 | 4.89E-07 | 5.86E-05 | 203.184 | 3.475126 | 0.557383 | 6.234713 | 4.53E-10 | 3.68E-07 |
| S100Z | 57.6843 | 3.009216 | 0.825406 | 3.645742 | 0.000267 | 0.01151 | 43.54291 | 3.405676 | 0.804902 | 4.23117 | 2.32E-05 | 0.002611 |
| SCIMP | 394.9548 | 2.352425 | 0.457214 | 5.145127 | 2.67E-07 | 3.51E-05 | 264.1773 | 2.601252 | 0.50076 | 5.194609 | 2.05E-07 | 6.25E-05 |
| SDPR | 472.0434 | 4.686411 | 0.480471 | 9.753786 | 1.78E-22 | 4.66E-19 | 242.15 | 4.504387 | 0.518832 | 8.681784 | 3.90E-18 | 1.58E-14 |
| SLC1A3 | 766.4659 | 2.124104 | 0.420645 | 5.04964 | 4.43E-07 | 5.38E-05 | 467.9515 | 2.244331 | 0.471737 | 4.757593 | 1.96E-06 | 0.000385 |
| SLC38A1 | 104.3857 | 2.440307 | 0.641981 | 3.801217 | 0.000144 | 0.007158 | 69.15852 | 2.665786 | 0.650068 | 4.100779 | 4.12E-05 | 0.004082 |
| SOCS1 | 299.915 | 2.938811 | 0.487045 | 6.03396 | 1.60E-09 | 4.57E-07 | 146.0481 | 2.698574 | 0.604153 | 4.466704 | 7.94E-06 | 0.001167 |
| SPIB | 61.62734 | 2.734368 | 0.817948 | 3.342961 | 0.000829 | 0.027512 | 35.57334 | 2.74525 | 0.753331 | 3.644148 | 0.000268 | 0.016692 |
| SPNS3 | 72.62651 | 3.738949 | 0.817695 | 4.572548 | 4.82E-06 | 0.000422 | 31.71648 | 3.335768 | 0.871835 | 3.826147 | 0.00013 | 0.009921 |
| SPON2 | 61.8592 | 4.158354 | 0.938491 | 4.430895 | 9.38E-06 | 0.000737 | 30.78417 | 3.928281 | 0.897263 | 4.37807 | 1.20E-05 | 0.00157 |
| SRGAP2 | 697.478 | 1.464587 | 0.459742 | 3.18567 | 0.001444 | 0.042119 | 432.0292 | 1.640071 | 0.435798 | 3.763375 | 0.000168 | 0.011614 |
| SYTL1 | 98.69445 | 4.09315 | 0.748258 | 5.470238 | 4.49E-08 | 7.97E-06 | 38.11527 | 3.483037 | 0.796506 | 4.372896 | 1.23E-05 | 0.001584 |
| TBC1D10C | 161.9828 | 2.375687 | 0.579715 | 4.098023 | 4.17E-05 | 0.002544 | 74.39713 | 2.044422 | 0.585073 | 3.4943 | 0.000475 | 0.026052 |
| TCF7L2 | 396.3097 | 2.507124 | 0.479753 | 5.225865 | 1.73E-07 | 2.39E-05 | 183.8682 | 2.189114 | 0.533701 | 4.101758 | 4.10E-05 | 0.004082 |
| TMEM156 | 68.0621 | 3.641909 | 0.865651 | 4.207134 | 2.59E-05 | 0.00175 | 42.16444 | 3.744279 | 0.8345 | 4.486852 | 7.23E-06 | 0.001104 |
| TRAC | 102.1663 | 2.780947 | 0.712582 | 3.902634 | 9.52E-05 | 0.004955 | 55.70403 | 2.719854 | 0.705879 | 3.853147 | 0.000117 | 0.009175 |
| TRAF3IP3 | 360.0331 | 2.105914 | 0.460989 | 4.568252 | 4.92E-06 | 0.000427 | 192.8379 | 2.020128 | 0.524078 | 3.854628 | 0.000116 | 0.009175 |
| TRBC1 | 99.79343 | 2.271431 | 0.657111 | 3.456694 | 0.000547 | 0.019555 | 60.56893 | 2.384084 | 0.643524 | 3.704731 | 0.000212 | 0.013511 |
| TRBC2 | 154.1551 | 3.493104 | 0.662635 | 5.271535 | 1.35E-07 | 1.95E-05 | 65.65652 | 3.049928 | 0.68795 | 4.43336 | 9.28E-06 | 0.001271 |
| TSC22D3 | 7796.404 | 2.865459 | 0.40375 | 7.097111 | 1.27E-12 | 6.19E-10 | 3845.435 | 2.639833 | 0.484599 | 5.447454 | 5.11E-08 | 1.84E-05 |
| TSPAN32 | 112.4071 | 2.807719 | 0.643829 | 4.360971 | 1.29E-05 | 0.000994 | 68.31171 | 2.894787 | 0.654905 | 4.420163 | 9.86E-06 | 0.001322 |
| U1 | 70.95977 | 3.600194 | 0.785908 | 4.580934 | 4.63E-06 | 0.00041 | 53.33587 | 3.986653 | 0.736625 | 5.412055 | 6.23E-08 | 2.17E-05 |
| VSIR | 2196.219 | 2.170421 | 0.39951 | 5.432714 | 5.55E-08 | 9.46E-06 | 1187.25 | 2.099969 | 0.438022 | 4.794205 | 1.63E-06 | 0.000327 |
| WFDC21P | 43.87937 | 9.31366 | 2.218172 | 4.1988 | 2.68E-05 | 0.001797 | 27.41513 | 9.389534 | 2.233447 | 4.204054 | 2.62E-05 | 0.00278 |
| ZBTB16 | 331.0318 | 3.710818 | 0.497955 | 7.452114 | 9.19E-14 | 5.48E-11 | 313.0433 | 4.432609 | 0.652124 | 6.797187 | 1.07E-11 | 1.08E-08 |
| ZFAND5 | 4427.237 | 2.505081 | 0.374495 | 6.689229 | 2.24E-11 | 8.92E-09 | 1627.139 | 1.826465 | 0.411245 | 4.441303 | 8.94E-06 | 0.001239 |
| ZFP36L2 | 6471.316 | 1.568577 | 0.395063 | 3.970451 | 7.17E-05 | 0.003956 | 3233.958 | 1.393636 | 0.407276 | 3.421849 | 0.000622 | 0.032004 |
| ZNF812P | 215.1904 | 5.692933 | 0.7539 | 7.551307 | 4.31E-14 | 2.69E-11 | 96.68879 | 5.319723 | 0.722961 | 7.358243 | 1.86E-13 | 3.25E-10 |
