# Supplementary material for: Key Cell Types and Biomarkers in Heart Failure Identified through Analysis of Single-Cell and Bulk RNA Sequencing Data
Source: Mediators Inflamm. 2023 Dec 26;2023:8384882. doi: 10.1155/2023/8384882 (PMC10761229; doi:10.1155/2023/8384882)
Supplement: Supplementary 3 — Table S1: GO biological process annotation of CXCR4, CD74, and MIF. [file 8384882.f3.docx]

Table S1 GO biological process annotation of CXCR4, CD74 and MIF.

| GO | Description | Count | Log10 (p) | Log10(q) |
| --- | --- | --- | --- | --- |
| GO:0050920 | regulation of chemotaxis | 3 | -6.36 | -2.01 |
| TRR01158 | Regulated by: RELA | 3 | -5.90 | -1.90 |
| TRR00875 | Regulated by: NFKB1 | 3 | -5.90 | -1.90 |

Note: p is the p-value; q is the adjust p-value
